# Artificial Intelligence-Aided Diagnosis Software to Identify Highly Suspicious Pulmonary Nodules

**DOI:** 10.3389/fonc.2021.749219

**Published:** 2022-02-15

**Authors:** Jun Lv, Jianhui Li, Yanzhen Liu, Hong Zhang, Xiangfeng Luo, Min Ren, Yufan Gao, Yanhe Ma, Shuo Liang, Yapeng Yang, Zhenchun Song, Guangming Gao, Guozheng Gao, Yusheng Jiang, Ximing Li

**Affiliations:** ^1^ Medical Radiology Department, Tianjin Chest Hospital, Tianjin, China; ^2^ LinkDoc Technology, Beijing, China; ^3^ Tianjin Cardiovascular Institute, Tianjin Chest Hospital, Tianjin, China; ^4^ Academy of Medical Engineering and Translational Medicine, Tianjin University, Tianjin, China; ^5^ Pathology Department, Tianjin Chest Hospital, Tianjin, China

**Keywords:** artificial intelligence-assisted diagnosis, low-dose radiation, pulmonary nodules, spiral computed tomography, target scan

## Abstract

**Introduction:**

To evaluate the value of artificial intelligence (AI)-assisted software in the diagnosis of lung nodules using a combination of low-dose computed tomography (LDCT) and high-resolution computed tomography (HRCT).

**Method:**

A total of 113 patients with pulmonary nodules were screened using LDCT. For nodules with the largest diameters, an HRCT local-target scanning program (combined scanning scheme) and a conventional-dose CT scanning scheme were also performed. Lung nodules were subjectively assessed for image signs and compared by size and malignancy rate measured by AI-assisted software. The nodules were divided into improved visibility and identical visibility groups based on differences in the number of signs identified through the two schemes.

**Results:**

The nodule volume and malignancy probability for subsolid nodules significantly differed between the improved and identical visibility groups. For the combined scanning protocol, we observed significant between-group differences in subsolid nodule malignancy rates.

**Conclusion:**

Under the operation and decision of AI, the combined scanning scheme may be beneficial for screening high-risk populations.

## Introduction

In 2018, the American Cancer Society reported that the worldwide incidence and mortality rates were higher for lung cancer than for any other form of cancer (1). Early diagnosis and treatment are essential for improving survival and reducing mortality among patients with lung cancer. Although low-dose computed tomography (LDCT) screening for lung cancer has been validated in several randomised controlled trials (2, 3), this method is not without limitations, including a high false-positive rate (2). In clinical practice, the high false-positive rate is related to the relatively poor ability of LDCT to reveal fine structural details within lesions, compared to high-resolution computed tomography (HRCT) (4). For many patients with positive LDCT findings, diagnostic evaluations may yield uncertain results, in turn leading to uncertainty regarding long-term treatment strategies, increasing patient anxiety and resulting in an inefficient allocation of medical resources (5).

HRCT (4) can significantly improve the visualisation of fine structural details. Thus, LDCT, combined with HRCT of local lung nodules, may compensate for the deficiencies of LDCT in lung cancer screening. However, to our knowledge, no previous studies have investigated the types of lung nodules or patient subgroups in which this combined scanning strategy may be effective. Furthermore, the lack of a diagnostic protocol for combined scanning forces physicians to rely on subjective judgments when recommending HRCT.

Continued advancements in artificial intelligence (AI) may aid clinicians in detecting and diagnosing lung nodules (6, 7). In the present study, we utilised AI to assist in diagnosing lung nodules during LDCT. Lung nodules were analysed in real time *via* AI-assisted software, and the system recommended further local HRCT target scans in cases of highly suspicious nodules.

## Materials and Methods

**Figure 1:**
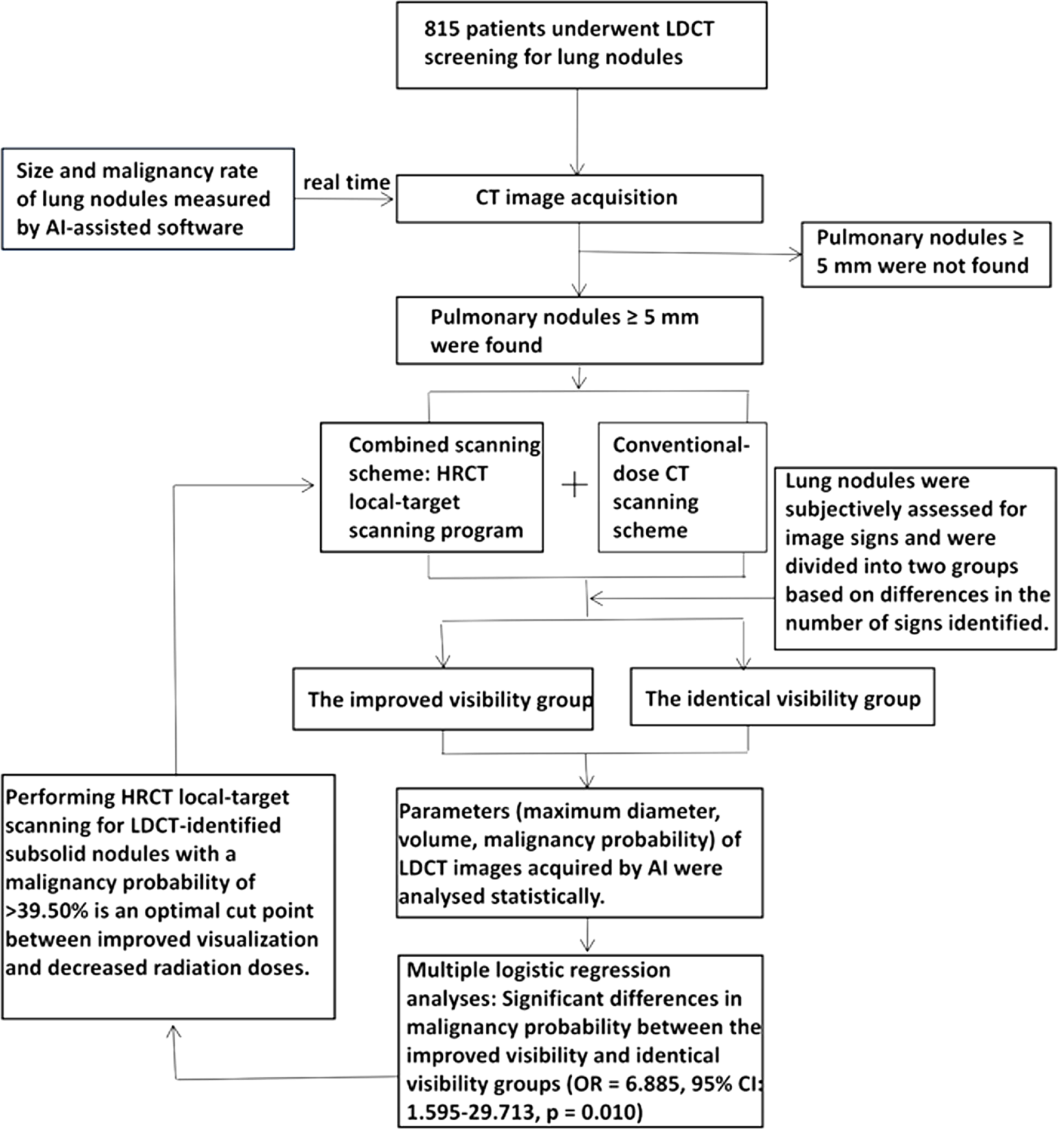
The flow chart shows the method designed to check the pulmonary nodules by AI calculation and to use the maximum diameter to carry out local targeted HRCT. In a later statistical analysis, we found that subsolid nodule and nodules with a malignant rate >39.50% were more visible with this method. We should determine whether to carry out local target scanning according to the malignant rate of subsolid nodules in order to guide the future LDCT screening with real-time AI pulmonary nodule detection.

**Figure 2:**
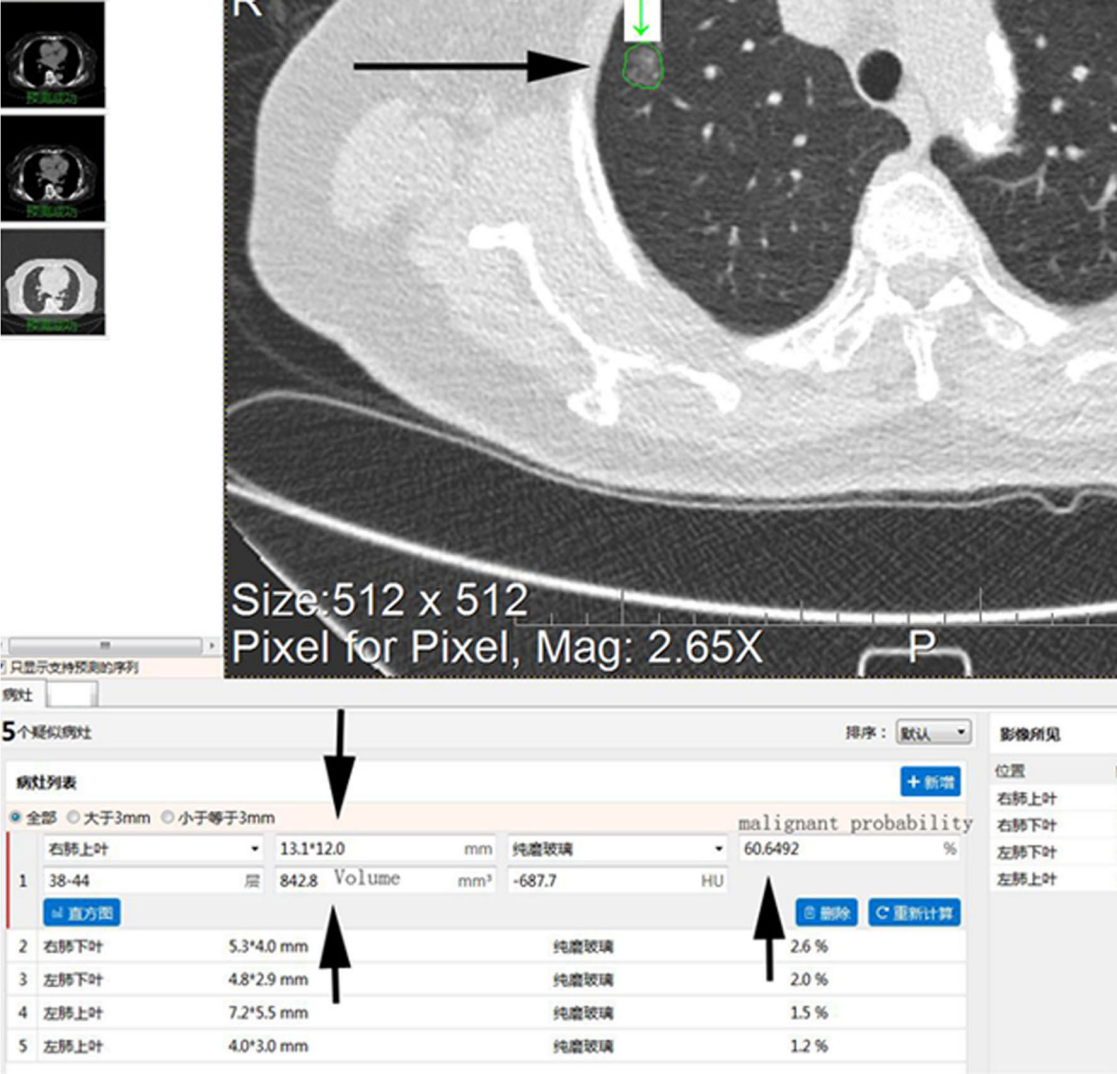
Screenshot of AI-assisted diagnostic software in the analysis of a specific CT image. The long arrow shows the analysis of an LDCT image in which all detected nodules have been marked. The short arrow indicates detailed information regarding the underlying nodule (e.g., location, maximum diameter, minimum diameter, density type, malignancy probability, volume, mean CT value). AI, artificial intelligence; LDCT, low-dose computed tomography; CT, computed tomography.

From September 2019 to December 2019, 815 patients underwent LDCT screening for lung nodules at our hospital. During inspiratory breath-hold acquisition, the apex to the bottom of the lungs was scanned. The images were observed using lung windows (window width: 1,600 HU, window level: −550 HU) and through the AI-assisted diagnostic system to determine the subsequent scanning scheme (**Figure 1**). AI calculated the LDCT images in real time, and the time was about two minutes. All the pulmonary nodules were found and sorted according to the maximum diameter of the nodules (**Figure 2**). These examiners further evaluated images from patients with lung nodules exhibiting a maximum diameter of ≥5 mm using the combined imaging strategy. According to this protocol, the entire lung was scanned using conventional-dose CT, and the largest lesions were scanned using local HRCT (**Supplementary Figure 1**). Patients with no lung nodules measuring ≥5 mm in diameter were scanned using LDCT only.

In this study, 113 patients exhibited pulmonary nodules with a maximum diameter of ≥5 mm (43 men, 70 women; mean age: 65.6 ± 8.54 years; age range: 50–82 years). The ethics committee of our institution approved the study. Patients provided written informed consent before the examination. The work described was carried out in accordance with the code of ethics of the World Medical Association (Declaration of Helsinki) for experiments involving humans.

### Scanning Parameters

Images were obtained using a multi-slice CT (MSCT) scanner (Philips Brilliance, iCT 256). The following parameters were used for LDCT scans: tube voltage, 100 kV; tube current, 30, 40, or 50 mAs based on body mass index (BMI) values for each patient (BMI <18.5 kg/m^2^, BMI ≤18.5–≤25 kg/m^2^, BMI >25 kg/m^2^); reconstruction, iDose4; filter, lung enhanced; field of view (FOV), 350 mm; layer spacing, 1.5 mm; layer thickness, 1.5 mm; pitch, 0.758; rotation time, 0.5 s; reconstruction matrix, 512 × 512. For conventional-dose CT scans of pulmonary nodules, the tube voltage was set to 120 kV, the tube current was set to 100–250 mAs and the standard reconstruction setting was used. All other parameters were the same as those used for LDCT. Parameters for HRCT target scanning were as follows: tube voltage, 120 kV; tube current, 190–330 mAs; reconstruction, iDose3; filter, Y-Sharp; FOV, 150 mm; pitch, 0.399; rotation time, 0.33 s. The remaining HRCT parameters were the same as those used for LDCT.

The AI-assisted diagnostic system for lung nodules is commercially available. The data were retrieved from the Luna, NSCL and LIDC open datasets, as well as from 18 cooperative hospitals. All data were used for training of the AI system. The detection effect was verified on the Luna dataset (area under the free-response receiver operating characteristic [ROC] curve, 0.8612), while benign and malignant rates were verified on the DSB 2017 dataset (area under the curve [AUC], 0.89). The AI system consists of two modules: a detection module and a segmentation module (8). The detection module outputs the predicted nodule with a 3-D bounding box and nodule malignancy. The backbone of the detection module is a 3-D U-Net, and the output feature map is passed to a region proposal network for bounding box and malignancy prediction (9). The network is trained by backpropagation with the stochastic gradient descent optimiser. Based on the bounding box, the region of interest of detected nodules is calculated and passed to the nodule segmentation module (10), which outputs a reconstructed 3-D nodule mask for each detected nodule. Morphological features, such as the longest diameter, shortest diameter and volume of the nodule with the largest cross-sectional area, are calculated based on the output 3-D mask.

All CT images were obtained by two physicians with 11 and 17 years of experience in diagnosing chest images, respectively. Then, the images were examined in a double-blinded manner by two experienced radiologists. A senior imaging physician with 17 years of experience provided the final diagnosis in case of a disagreement between the two radiologists. All lung nodules were categorised as solid or subsolid nodules (including pure ground-glass nodules [GGN] and part-solid GGNs) according to lesion density. CT images were subjectively evaluated based on the following features: 1) the presence of lobulations, defined as deeper notches at the edges of tumours; 2) the presence of spiculations, defined as fine, short, unbranched opacities radiating from the edges of the tumour to surrounding areas; 3) the presence of abnormal air bronchograms (11) (i.e. narrowing, dilation, tortuous or separation of bronchi within the nodule); 4) the presence of a necrotic cavity within the lesion and discharge of necrotic tissue through the bronchus; 5) the presence of air-containing spaces (12), reflecting the pathological expansion of the physiological cavity in the lung; 6) the presence of single or multiple bubble-like lucencies (13) (i.e. 1–2 mm dot-like translucent shadows within the lesion); 7) the presence of pleural indentation in the case of pleural involvement.

Patients for whom the difference between the number of signs on the target HRCT and the conventional image was >1 were included in the improved visibility group (i.e. more signs observed during target scanning). The remaining patients were assigned to the identical visibility group.

### Statistical Analyses

Stata version 14.1 (StataCorp, College Station, TX, USA) was used to perform statistical analyses. Continuous variables with a skewed distribution were expressed as medians and quartiles, while categorical data were expressed as rates or percentages. Wilcoxon rank-sum tests were used to compare continuous variables with skewed distributions. Kruskal–Wallis tests were used to compare data across multiple groups, while chi-square tests were used to compare data between groups. Univariate and multivariate logistic regression analyses were performed to determine the clear odds ratio (OR) of the target scan. All tests were two-tailed, and the level of statistical significance was set to a p value of <0.05. ROC curves were generated using GraphPad Prism 7.0 (GraphPad Software, Inc., La Jolla, CA, USA). GraphPad Prism 7.0 was also used to calculate the AUC and determine the optimal cut-off values for low-dose volume and malignancy rate.

## Results

Among the 815 physical examinations, 113 were selected for further screening. Total radiation doses were significantly lower for LDCT combined with local HRCT than for conventional-dose CT (358.93 vs. 473.67, p < 0.01) (**Supplementary Table 1**).

Among the 113 included cases, the largest lesions were classified as solid in 66 cases and subsolid in 47 cases. AI-assisted software was then used to determine the number of lung nodules detected *via* LDCT and conventional-dose CT. All lung nodules, not only the largest lesions detected by the software in the target scan, were divided into three groups based on maximum diameter: <5, 5–10 and >10 mm. Significantly fewer nodules with a diameter of <5 mm were detected by the AI system during the combined imaging scheme than during conventional-dose CT (p = 0.000) (**Supplementary Table 2**). However, no such differences were observed in the 5–10 or >10 mm groups (p > 0.05).

Following the aforementioned procedures, the AI-assisted software was used to identify the largest lesions in each of the 113 included patients (**Figure 2**). No significant differences in maximum diameter, volume or malignancy probability were observed between the combined scanning scheme and the conventional scanning scheme (p > 0.05) (**Supplementary Table 3**).

**Figure 3:**
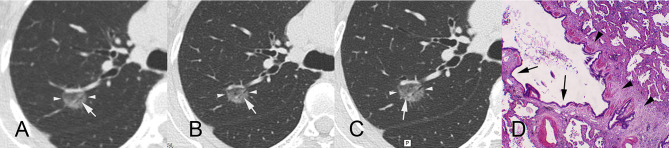
A 55-year-old female patient with ground-glass nodules in the posterior segment of the right upper lobe detected by LDCT screening. AI indicated that the malignant rate was 97.46%. Surgical resection revealed invasive mural adenocarcinoma. CT images reconstructed by lung window. **(A)** LDCT image. The white arrow indicates that there is an obscure air bronchogram in the nodule. **(B)** Conventional CT image. The white arrow indicates a slightly clear air bronchogram in the nodule. **(C)** HRCT local-target scanning image. An abnormal air bronchogram is visible. The white arrow indicates that the target scan shows a finer tortuous bronchial sign. **(D)** Pathological HE staining image (×40) showing diffuse tumour tissue around bronchioles, infiltration and growth of tumour tissue, and proliferation of fibrous connective tissue (arrowheads), resulting in bronchiectasis (arrows). AI, artificial intelligence; LDCT, low-dose computed tomography; CT, computed tomography; HRCT, high-resolution computed tomography.

**Figure 4:**
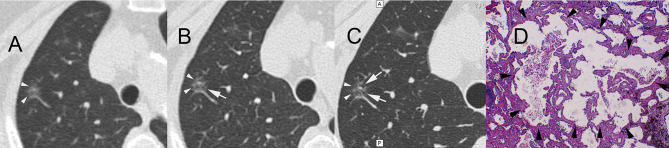
A 61-year-old female patient with ground-glass nodules in the posterior segment of the right upper lobe detected by LDCT screening. AI indicated that the malignant rate was 60.65%. Surgical resection revealed invasive mural adenocarcinoma. CT images reconstructed by lung window. **(A)** LDCT image. The white arrowheads indicate GGNs. **(B)** Conventional CT image. The white arrow indicates small bubble-like lucencies with unclear boundaries. **(C)** HRCT local-target scanning image. The white arrows indicate several bubble-like lucencies with clear boundaries. **(D)** Pathological HE staining image (×40) showing the destruction and fusion of two bubble-like lucencies (arrowheads) in the middle of the background of invasion and growth of tumour tissue. AI, artificial intelligence; LDCT, low-dose computed tomography; GGN, ground-glass nodule; HRCT, high-resolution computed tomography.

**Supplementary Table 4** shows the results of subjective evaluations of the largest lesions in the 113 patients. When the three scanning methods were compared for subsolid nodules, we observed significant differences in the presence of air bronchogram abnormalities (**Figure 3**) and bubble-like lucencies (**Figure 4**) (p < 0.05).

**Figure 5:**
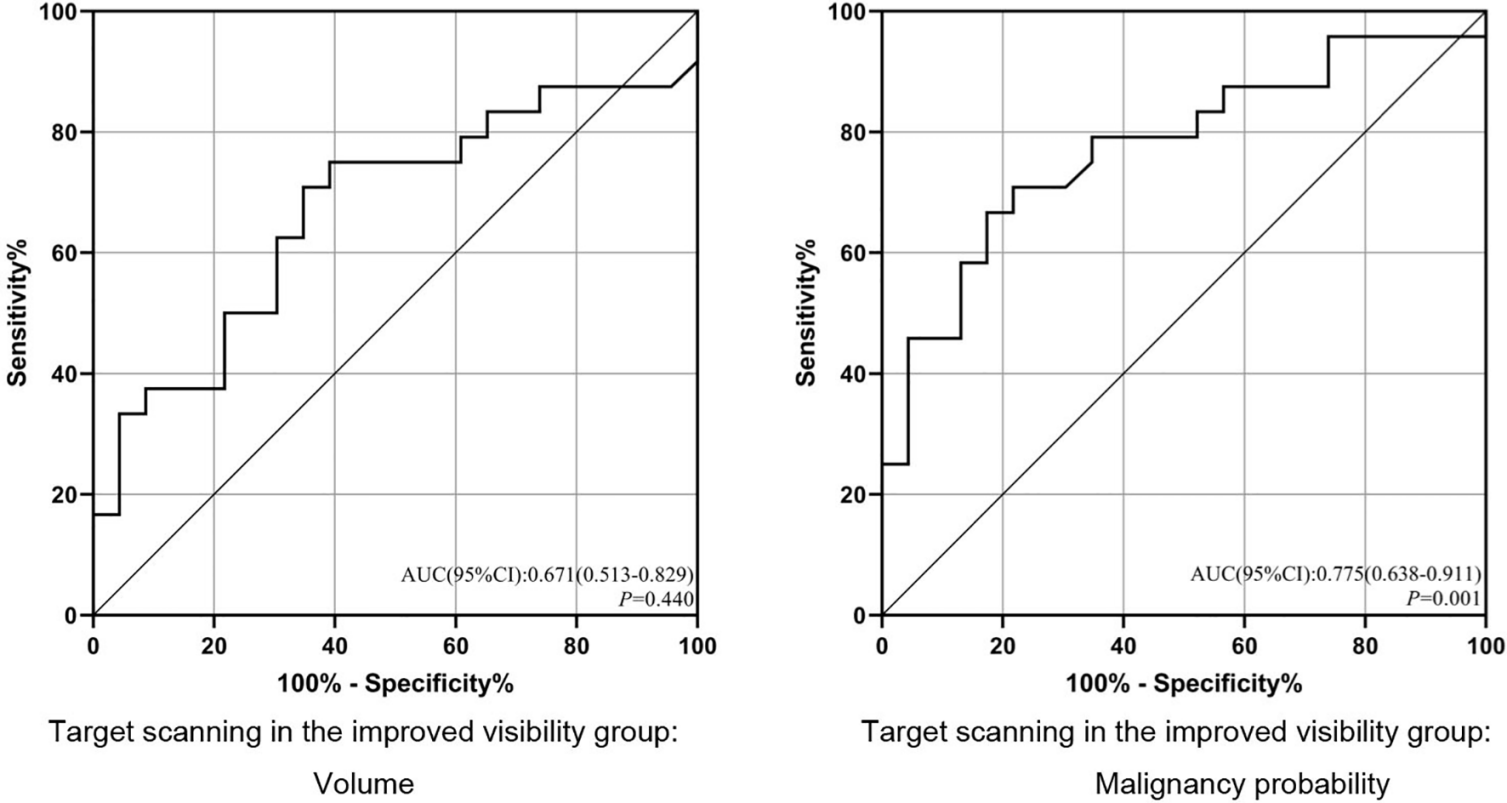
ROC analysis of the prediction model for improved visibility. ROC, receiver operating characteristic; AUC, area under the ROC curve. p < 0.05.

For solid nodules, our single-factor analysis revealed no statistically significant differences in maximum diameter, volume or malignancy probability between the improved visibility and identical visibility groups (**Table 1**). In addition, we observed no significant differences in the maximum diameter of subsolid nodules between the improved visibility and identical visibility groups. However, for subsolid nodules, there were significant differences in volume (474.54 [283.19, 1,599.08] vs. 306.40 [171.40, 489.26] mm^3^, p < 0.05) and malignancy probability (55.96% vs. 12.8%, p < 0.01) between the improved visibility and identical visibility groups. The classification model based on volume and malignancy probability accurately predicted whether the local-target scanning *via* HRCT could improve the visibility of subsolid nodules (**Figure 5**).

**Table 1:** Comparisons between the improved visibility and identical visibility groups.

Solid nodules	Improved visibility group (n = 8) [median (Q_25,_ Q_75_)]	Identical visibility group (n = 58) [median (Q_25,_ Q_75_)]	Z/X^2^	p-value
Maximum diameter	7.47 (6.55, 11.18)	8.25 (6.90, 11.58)	-0.678	0.498
Volume	202.40 (122.90, 783.65)	227.00 (124.18, 472.35)	-0.236	0.814
Malignancy probability	2.48 (1.57, 6.55)	2.14 (0.80, 4.88)	-0.825	0.409
**Subsolid nodules**	**Improved visibility group (n = 24)**	**Identical visibility group (n = 23)**	**Z/X^2^**	**p-value**
Maximum diameter	10.30 (8.70, 16.79)	8.71 (8.02, 12.81)	-1.532	0.125
Volume	474.54 (283.19, 1599.08)	306.40 (171.40, 489.26)	-2.011	0.044
Malignancy probability	55.96 (21.35, 71.03)	12.80 (2.32, 32.30)	-3.224	0.001

Multiple logistic regression analyses (**Table 2**) yielded cut-off values of >347.7 mm^3^ for subsolid nodule volume and >39.50% for malignancy rates. For the combined scanning scheme, we observed significant differences in malignancy probability between the improved visibility and identical visibility groups (OR = 6.885, 95% confidence interval (CI): 1.595–29.713, p = 0.010). For a malignancy probability of >39.50%, sensitivity and specificity values were 66.67% and 82.61%, respectively.

**Table 2:** Multivariate analysis of subsolid nodule findings in the improved visibility and identical visibility groups.

Variable	p-value	OR	95% CI for OR	
			Lowerbound	Upperbound
Volume, >347.7 m^3^	0.254	2.271	0.555	9.294
Malignancy probability, >39.50%	0.010	6.885	1.595	29.713

OR, odds ratio; CI, confidence interval.

## Discussion

Previous studies offer evidence showing that in selected high-risk groups, LDCT examination can significantly reduce lung cancer mortality (14, 15). During LDCT, radiation dosages are reduced by decreasing the tube current and voltage. However, decreasing the X-ray doses also affects the signal-to-noise ratio, in turn impacting the visualisation of fine structures within lung nodules. The ability to visualise certain fine structures within lung nodules is essential for an accurate diagnosis.

Previous LDCT studies (15, 16) have focused on optimising the risk-reward ratio by reducing tube current or improving image reconstruction algorithms. However, such developments have been associated with only marginal improvements in the assessment of malignancy in high-risk nodules, as accurate diagnosis requires high-resolution images, which cannot be obtained *via* LDCT. In contrast to LDCT and conventional-dose CT, HRCT allows the visualisation of microstructures within high-risk nodules. Indeed, we observed a significant difference in the presence of air bronchogram abnormalities and bubble-like lucencies in subsolid nodules between the LDCT and HRCT conditions (p < 0.05). Compared with conventional CT local-target reconstruction, HRCT target scanning has several advantages, such as small geometric distortion, improved density and spatial resolution, higher contrast and sharper edges of tiny structures, due to its unique algorithm (4, 17). The imager can obtain more information when observing the microscopic structure of minor lesions on account of the abovementioned advantages of HRCT target scanning. However, considering the above-average radiation doses (4), chest HRCT is not recommended in low-risk cases. In the present study, we utilised a combined LDCT/HRCT scanning strategy to investigate highly suspicious lung nodules identified *via* an AI-assisted system. Our combined scanning strategy of using AI-assisted diagnostic software to target high-risk locations systematically during HRCT represents an ideal approach in which excessive radiation doses are allocated to high-risk locations only and achieves the best possible trade-off between accuracy/specificity and total radiation dose.

In the present study, total radiation doses were significantly lower for the AI-optimised combined scanning scheme than for the conventional scanning scheme (p < 0.01), suggesting that the combined scanning scheme is no more harmful to the patient than conventional-dose scanning. In addition, the number of nodules measuring <5 mm in diameter detected by the AI-assisted software was significantly lower in the LDCT condition than in the conventional-dose condition (p < 0.01). This finding suggests that, due to the low quality of the images produced by LDCT, the sensitivity of the AI-assisted software for detecting nodules measuring <5 mm in diameter was reduced, consistent with the results of visual examination. However, we observed no significant differences in detection sensitivity for nodules of 5–10 or >10 mm between LDCT and conventional-dose scanning (p > 0.05). Considering that previous studies have reported that nodules with diameters of <5 mm do not warrant a long-term follow-up (18), reduced sensitivity for these nodules is less likely to affect clinical diagnosis. Furthermore, we observed no significant differences in maximum diameter, volume or malignancy rates between the combined and conventional scanning strategies for nodules measuring 5–10 and >10 mm in diameter (p > 0.05). These results indicated that our AI-optimised combined scanning strategy is likely to achieve performance similar to conventional scanning.

The present study also investigated the types of nodules for which HRCT is sensitive. In this case, we defined ‘sensitivity’ as the ability of HRCT to identify more clinical characteristics of subsolid nodules, including the presence of air bronchograms and bubble-like lucencies. The present study was based on the underlying principle that additional radiation doses should be allocated to locations where HRCT can reveal significantly more information than LDCT or conventional-dose scanning. When there was insufficient statistical evidence to suggest that HRCT could yield such information, we opted to perform local HRCT on those locations. For the 66 solid nodules, we observed no significant difference in maximum diameter, volume or malignancy probability (p > 0.05). Solid nodules rarely exhibit hollow microstructures such as abnormal air bronchograms, bubble-like lucencies or air-containing spaces, considering their density. In addition, boundary-related features, such as lobulations, spiculations and pleural indentation signs, can be detected using conventional CT in cases wherein the amount of information gained using HRCT is insignificant. In contrast, subsolid nodules are correlated with the presence of hollow microstructures.

Consistent with the findings of previous studies, our results indicated that HRCT was associated with significantly better visualisation of these hollow microstructures than conventional CT (4). The results of our multivariate analysis indicated that a malignancy probability of >39.50% for subsolid nodules was associated with improved visibility on HRCT (subjective visual information gain: 6.885 x [p < 0.05], sensitivity: 66.67%, specificity: 82.61%). Our results suggest that HRCT local-target scanning should be recommended when AI-assisted LDCT identifies a subsolid nodule with a malignancy probability of >39.50%. HRCT local-target scanning is not recommended in these cases, considering the limited amount of information gained for other types of nodules.

The present study has some limitations of note. First, our investigation was not based on solid pathological evidence; interpretations of HRCT images were based on subjective assessments by physicians, and the estimated cut-off values determined using proprietary AI-assisted software were dependent on the version of the software. Second, our study included a small sample of 113 patients, necessitating larger follow-up studies to verify our findings.

Our combined scanning strategy was associated with significant decreases in radiation dose relative to conventional scanning, without significant changes in detection count, size or malignancy probability for high-risk nodules (nodules measuring ≥5 mm in diameter). Furthermore, our results demonstrate that performing HRCT local-target scanning for LDCT-identified subsolid nodules with a malignancy probability of >39.50% provides the optimal balance between improved visualisation and decreased radiation doses. This combined strategy may be suitable for the practical application of pulmonary nodule screening.

In summary, the combined scanning scheme is conducive to screening high-risk pulmonary nodules in the population. The application of AI can predict in which pulmonary nodules to implement this scheme, which is highly targeted and has more advantages than previous subjective evaluation. In addition, the application of AI can reduce unnecessary radiation doses and improve the visibility of fine structure within small nodules.

## Data Availability Statement

The original contributions presented in the study are included in the article/**Supplementary Material**. Further inquiries can be directed to the corresponding author.

## Ethics Statement

The studies involving human participants were reviewed and approved by Medical Ethics Committee of Tianjin Chest Hospital. The patients/participants provided their written informed consent to participate in this study.

## Author Contributions

Guarantor of integrity of the entire study: JuL. Study concept and design: HZ. Literature research: JiL, ZS, and YG. Clinical studies: GZG, SL and XML. Experimental studies/data analysis: GMG and YY. Statistical analysis: MR. Manuscript preparation: YJ and YL. Manuscript editing: XFL and YM. All authors contributed to the article and approved the submitted version.

## Funding

This project was supported by Grant No. 18ZXZNSY00400 from the Tianjin Science and Technology Plan Project on the construction of data platforms for artificial intelligence-assisted diagnosis of lung nodules and clinical applications of such systems in the diagnosis and treatment of lung cancer.

## Conflict of Interest

Authors XFL, GMG and YJ were employed by company LinkDoc Technology.

The remaining authors declare that the research was conducted in the absence of any commercial or financial relationships that could be construed as a potential conflict of interest.

## Publisher’s Note

All claims expressed in this article are solely those of the authors and do not necessarily represent those of their affiliated organizations, or those of the publisher, the editors and the reviewers. Any product that may be evaluated in this article, or claim that may be made by its manufacturer, is not guaranteed or endorsed by the publisher.

## Supplementary Material

Supplementary Figure 1 The left side of the image is the whole lung field LDCT scan (arrows), and the right side is the local HRCT scan for the largest lesion (arrowheads). Total radiation doses were significantly lower for LDCT combined with local HRCT than for conventional-dose CT (358.93 vs 473.67, p < 0.01, n=113).
