# Supplementary figures and images for: Artificial Intelligence-Aided Diagnosis Software to Identify Highly Suspicious Pulmonary Nodules
Source: Front Oncol. 2022 Feb 15;11:749219. doi: 10.3389/fonc.2021.749219 (PMC8886673; doi:10.3389/fonc.2021.749219)

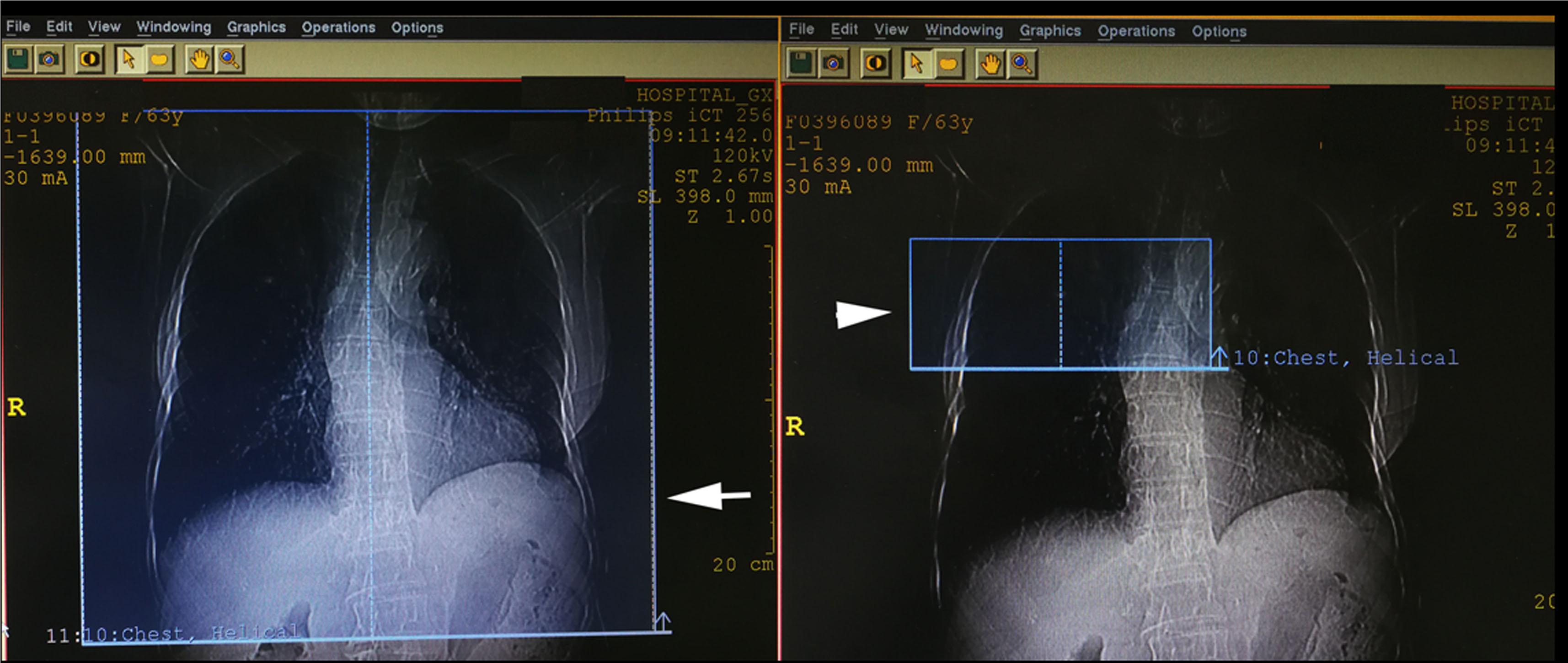

Supplement: Supplementary Figure 1 — The left side of the image is the whole lung field LDCT scan (arrows), and the right side is the local HRCT scan for the largest lesion (arrowheads). Total radiation doses were significantly lower for LDCT combined with local HRCT than for conventional-dose CT (358.93 vs 473.67, p < 0.01, n=113). [file Image_1.jpeg]
