# Supplementary material for: Artificial Intelligence-Aided Diagnosis Software to Identify Highly Suspicious Pulmonary Nodules
Source: Front Oncol. 2022 Feb 15;11:749219. doi: 10.3389/fonc.2021.749219 (PMC8886673; doi:10.3389/fonc.2021.749219)
Supplement: Supplementary file 2 [file Table_1.docx]

**Table S1.** Comparison of radiation doses between LDCT, HRCT target scanning, and conventional CT (n = 113)

|  | DLP (mGy-cm) mean ± SD | CTDIvol (mGy) mean ± SD |
| --- | --- | --- |
| Conventional | 473.67 ± 117.94 | 11.78 ± 2.73 |
| LDCT | 75.27 ± 9.53^*^ | 1.87 ± 0.22^*^ |
| HRCT | 286.19 ± 81.81^*, **^ | 17.11 ± 3.96^*, **^ |
| F-value | 650.725 | 873.865 |
| p-value | 0.000 | 0.000 |
| Conventional | 473.67 ± 117.94 |  |
| Combined | 358.93 ± 89.19 |  |
| t-value | 8.248 |  |
| p-value | 0.000 |  |

*Compared with conventional scanning, p < 0.05; **Compared with LDCT, p < 0.05. LDCT: low-dose computed tomography, HRCT: high-resolution computed tomography, CT: computed tomography, DLP: dose-length product, CTDIvol: volumetric computer tomography dose index
