# Supplementary material for: Artificial Intelligence-Aided Diagnosis Software to Identify Highly Suspicious Pulmonary Nodules
Source: Front Oncol. 2022 Feb 15;11:749219. doi: 10.3389/fonc.2021.749219 (PMC8886673; doi:10.3389/fonc.2021.749219)
Supplement: Supplementary file 3 [file Table_2.docx]

**Table S2**. Number of nodules with different diameters, detected by AI-assisted software, between LDCT and conventional CT scanning (n = 113)

| Nodules | <5 mm [median (Q_25,_ Q_75_)] | 5-10 mm [median (Q_25,_ Q_75_)] | >10 mm [median (Q_25,_ Q_75_)] | Total detected [median (Q_25,_ Q_75_)] |
| --- | --- | --- | --- | --- |
| LDCT | 2.00 (1.00, 4.00) | 5.00 (3.00, 8.00) | 1.00 (0.00, 1.00) | 9.00 (5.00, 13.00) |
| Conventional | 4.00 (2.00, 6.00) | 5.00 (3.00, 8.00) | 1.00 (0.00, 1.00) | 11.00 (6.00, 14.50) |
| Z-value | -6.277 | -0.652 | -0.225 | -5.428 |
| p-value | 0.000 | 0.514 | 0.822 | 0.000 |

AI: artificial intelligence; CT: computed tomography, LDCT: low-dose computed tomography
