# Supplementary material for: Artificial Intelligence-Aided Diagnosis Software to Identify Highly Suspicious Pulmonary Nodules
Source: Front Oncol. 2022 Feb 15;11:749219. doi: 10.3389/fonc.2021.749219 (PMC8886673; doi:10.3389/fonc.2021.749219)
Supplement: Supplementary file 4 [file Table_3.docx]

**Table S3.** Comparison of measurements and malignancy probability (n = 113)

|  | Solid nodules [median (Q_25,_ Q_75_)] | | | Subsolid nodules [median (Q_25,_ Q_75_)] | | |
| --- | --- | --- | --- | --- | --- | --- |
|  | Maximum diameter | Volume | Malignancy probability | Maximum diameter | Volume | Malignancy probability |
| Conventional | 8.20 (6.75, 10.77) | 213.85 (124.55, 435.45) | 2.80 (1.13, 8.31) | 9.00 (7.79, 13.12) | 266.10 (156.10, 585.02) | 33.90 (4.65, 68.80) |
| LDCT | 8.15 (6.88, 11.33) | 227.00 (126.65, 540.95) | 2.39 (0.89, 5.25) | 9.55 (8.15, 13.82) | 351.30 (209.90, 620.34) | 22.30 (4.46, 65.20) |
| HRCT | 8.25 (6.38, 11.30) | 205.78 (125.83, 429.55) | 4.64 (1.23, 8.87) | 10.08 (8.21, 14.41) | 288.90 (175.20, 732.22) | 33.86 (2.66, 75.67) |
| X^2^ | 0.518 | 0.452 | 4.623 | 1.543 | 2.370 | 0.128 |
| p-value | 0.772 | 0.798 | 0.099 | 0.462 | 0.306 | 0.938 |

LDCT: low-dose computed tomography, HRCT: high-resolution computed tomography
