# Supplementary material for: Artificial Intelligence-Aided Diagnosis Software to Identify Highly Suspicious Pulmonary Nodules
Source: Front Oncol. 2022 Feb 15;11:749219. doi: 10.3389/fonc.2021.749219 (PMC8886673; doi:10.3389/fonc.2021.749219)
Supplement: Supplementary file 5 [file Table_4.docx]

**Table S4.** Comparison of the number of fine structures observed in different scanning modes [n (%)]

|  |  | Lobulations  [n (%)] | Spiculations  [n (%)] | Abnormal air bronchogram  [n (%)] | Air-containing spaces  [n (%)] | Bubble-like lucencies  [n (%)] | Cavities  [n (%)] | Pleural indentation signs  [n (%)] |
| --- | --- | --- | --- | --- | --- | --- | --- | --- |
| Solid  nodules | Conventional | 11 (31.4) | 11 (32.4) | 4 (30.8) | () | () | () | 4 (36.4) |
|  | LDCT | 11 (31.4) | 9 (26.5) | 3 (23.1) | () | () | () | 3 (27.3) |
|  | HRCT | 13 (37.1) | 14 (41.2) | 6 (46.2) | () | () | () | 4 (36.4) |
|  | X^2^ | 0.278 | 1.349 | 1.153 |  |  |  | 0.204 |
|  | p-value | 0.870 | 0.509 | 0.562 |  |  |  | 0.903 |
| Subsolid nodules | Conventional | 15 (33.0) | 10 (33.3) | 9 (30.0) | 2 (22.2) | 4 (23.5) | 1 (33.3) | 8 (33.3) |
|  | LDCT | 12 (27.3) | 7 (23.3) | 5 (16.7) | 1 (11.1) | 2 (11.8) | 1 (33.3) | 6 (25.0) |
|  | HRCT | 17 (38.6) | 13 (43.3) | 16 (53.3)^*^ | 6 (66.7) | 11 (64.7)^*^ | 1 (33.3) | 10 (41.7) |
|  | X^2^ | 1.255 | 2.286 | 7.876 | 4.985 | 8.963 | 0.000 | 1.205 |
|  | p-value | 0.534 | 0.319 | 0.019 | 0.083 | 0.011 | 1.000 | 0.547 |

*Compared with LDCT, p < 0.05. LDCT: low-dose computed tomography, HRCT: high-resolution computed tomography
